# Proteoglycan-depleted regions of annular injury promote nerve ingrowth in a rabbit disc degeneration model

**DOI:** 10.1515/med-2021-0363

**Published:** 2021-11-02

**Authors:** Long Xin, Weixin Xu, Jian Wang, Fang Yu, Shunwu Fan, Xinwei Xu, Yang Yang

**Affiliations:** Department of Spine Surgery, Tongde Hospital of Zhejiang Province, Hangzhou 310012, Zhejiang Province, China; Department of Spine Surgery, The Affiliated Sir Run Run Shaw Hospital, Zhejiang University, Hangzhou 310020, Zhejiang Province, China; Department of Spine Surgery, Tongde Hospital of Zhejiang Province, Hangzhou 310012, Zhejiang Province, China; Department of Spine Surgery, Tongde Hospital of Zhejiang Province, No. 234 Gucui Road, Hangzhou 310012, Zhejiang Province, China

**Keywords:** PLGA/fibrin scaffold, chondroitinase ABC, proteoglycan, nerve ingrowth, disc degeneration

## Abstract

**Background:**

To assess the effects of proteoglycan-depleted regions of annular disruptions on nerve ingrowth in the injury site *in vivo*.

**Methods:**

New Zealand white rabbits (*n* = 18) received annular injuries at L3/4, L4/5, and L5/6. The experimental discs were randomly assigned to four groups: (a) an annular defect was created; (b) an annular defect implanted with a poly lactic-co-glycolic acid (PLGA)/fibrin/PBS plug; (c) an annular defect implanted with a PLGA/fibrin/chondroitinase ABC (chABC) plug; and (d) an uninjured L2/3 disc (control). Disc degeneration was evaluated by radiography, MRI, histology, and analysis of the proteoglycan (PG) content. Immunohistochemical detection of nerve fibers and chondroitin sulfate (CS) was performed.

**Results:**

The injured discs produced progressive and reliable disc degeneration. In the defective discs, the lamellated appearance of AF (Annulus fibrosus) was replaced by extensive fibrocartilaginous-like tissue formation outside the injured sites. In contrast, newly formed tissue was distributed along small fissures, and small blood vessels appeared in the outer part of the disrupted area in the PLGA/fibrin/PBS discs. More sprouting nerve fibers grew further into the depleted annulus regions in the PLGA/fibrin/chABC discs than in the control discs and those receiving PLGA/fibrin/PBS. In addition, the innervation scores of the PLGA/fibrin/chABC discs were significantly increased compared with those of the PLGA/fibrin/PBS discs and defected discs.

**Conclusion:**

ChABC-based PLGA/fibrin gel showed promising results by achieving biointegration with native annulus tissue and providing a local source for the sustained release of active chABC. Disc-derived PG-mediated inhibition of nerve and blood vessel ingrowth was abrogated by chABC enzymatic deglycosylation in an annular-injured rabbit disc degeneration model.

## 1 Introduction

Low back pain is an extremely common socioeconomic problem in orthopedics. Although the pathophysiology of this condition remains uncertain, degeneration of the intervertebral disc (IVD) is believed to be a major cause of low back pain [1,2,3]. This degenerative change ultimately results in increased matrix degradation, proinflammatory cytokine expression, and inferior mechanical properties [4,5,6]. More recently, progressive neurovascular growth in annular fissures and sensitization of nerve fibers have been observed in the degenerated IVD [7,8]. Ingrowth of nociceptive nerve fibers deep within disrupted IVDs is believed to be a potential source of discogenic pain in clinical cases [9,10,11,12].

It has been shown that aggrecan, a proteoglycan (PG) found in the disc, may act as a barrier to inhibit nerve fiber growth *in vivo* and *in vitro* [13,14,15,16]. However, the precise inhibitory mechanism of aggrecan remains poorly understood. In previous work, we established a minimally invasive annulotomy-induced rabbit model and demonstrated the growth of orientated growth-permissive nerves into annulus defects during the degenerative process. In addition, loss of PG at the injury site is involved in IVD degeneration and facilitates the infiltration of blood vessels and nerve fibers into disrupted disc tissue [17,18].

Chondroitinase ABC (chABC ) is a well-studied bacteria-derived enzyme that specifically depolymerizes glycosaminoglycan (GAG) side chains, inducing mild disc degeneration and favoring axonal regeneration by reducing chondroitin sulfate proteoglycan (CSPG) at the injury site [19,20,21,22,23,24]. According to our previous work, PLGA/fibrin constructs as a delivery system exhibit good biocompatibility, safety, and biodegradability [18,25], and promote tissue repair without compromising the function of the incorporated proteins [26,27,28,29,30]. Therefore, as an ideal carrier of chABC, PLGA/fibrin constructs represent a simple and desirable alternative to achieve local delivery in a controlled manner. In this system, cleavage of GAGs via digestion overcomes CSPG-mediated inhibition at the injured site of the degenerated IVD.

In the present study, we hypothesized that PG-depleted regions of the annular disruptions could facilitate nerve and blood vessel ingrowth into the deeper parts of the degenerated disc in a rabbit animal model. Here fibrin-based polymeric carriers loaded with chABC were implanted into the injured annular site to provide sustained local delivery *in vivo*, and the aim of this study was thus to assess the effects of degrading CSPG on neurite ingrowth during IVD degeneration.

## 2 Methods and materials

### 2.1 Preparation of PLGA-coated fibrin gel/ChABC constructs

PLGA sponges were fabricated using a porogen leaching method as reported previously [31]. The PLGA/fibrin/chABC constructs were prepared as follows. Fibrinogen was isolated from fresh human plasma, and the final concentration used in all experiments was based on previously established protocols [25,32]. ChABC (2U, Sigma, St. Louis, MO) was dissolved in buffer with a final concentration of 0.10 U/mL according to the product’s instructions and as previously reported [19,33]. Thereafter, fibrinogen (20 mg/mL) and chABC (0.10 U/mL) solutions were homogenized and sterilized by filtering through syringe filters. PLGA plugs (1.8 mm diameter and 4 mm length) were immersed into the homogeneous fibrinogen/chABC solution (1 mL) under reduced pressure. Composite constructs were lyophilized, imaged using a SEM, and subsequently stored at −20°C until further use.

### 2.2 Evaluation of chABC release from PLGA/fibrin *in vitro*

To obtain release profiles for each PLGA/fibrin sample, a gel loaded with chABC was suspended in 100 μL of PBS (pH 7.4) in polypropylene tubes placed in a shaker bath (37°C) at 100 rpm. Then, 10 μL of the supernatant was collected from each PLGA/fibrin sample at predetermined time intervals (1, 2, 3, 4, 5, 7, 9, 12 and 14 days) and individually stored at −20°C. According to a previously reported protocol [34], the enzymatic activity of the solution was assessed by measuring the formation of unsaturated disaccharides via chondroitin sulfate (CS) degradation over time (Sigma enzymatic assay-EC 4.2.2.4), as per the manufacturer’s instructions. Finally, the percentage of sample absorbance relative to the standard was calculated, and the composite construct’s accumulated release kinetic curve was obtained.

### 2.3 Animal surgery

A total of 18 New Zealand rabbits (age 8.60 ± 1.25 months and weight 3.42 ± 0.16 kg) were supplied by the Laboratory Animal Center, Zhejiang Province. Our Institutional Animal Care and Use Committee approved all aspects of the study. Rabbits were randomly allocated to 1-, 3-, and 6-month survival groups (*n* = 6 in each group). The surgical implantation procedure was described previously [17]. Consecutive levels of rabbit IVD, including L3/4, L4/5, and L5/6, were exposed using an anterolateral retroperitoneal approach. Annular injuries were randomly allocated to three disc levels: (1) in the annular defects group: annular defects (diameter 1.8 mm and 4 mm depth) were created using a minitrephine as previously described [17,18]; (2) in the PLGA/fibrin/PBS group: annular defects were filled with a PLGA/fibrin gel plug loaded with phosphate buffered saline; (3) in the PLGA/fibrin/chABC group: annular defects were filled with a PLGA/fibrin gel plug loaded with chABC; and (4) in the intact group: the L2/3 disc served as the uninjured control. Finally, the wound was closed in layers. Following surgery, rabbits were permitted free cage activity and food and water ad libitum.

### 2.4 Radiographic analyses and magnetic resonance imaging (MRI)

X-rays were obtained under general anesthesia (sodium pentobarbital, 30 mg/kg) at 1, 3, and 6 months after surgery (*n* = 6 per time point). The comparison of the disc height index (DHI) was based on the ratio of the injured disc height to the sum of the height of the two adjacent vertebral bodies as described previously [35,36]. DHI% values are expressed as DHI normalized to the baseline preoperative measurement (postoperative DHI/baseline DHI × 100). *In vivo* MRI of the lumbar spine was performed using a 1.5-T MRI system (Signa, General Electric, Milwaukee, Wisconsin). Midsagittal T2-weighted images were obtained for analysis as previously described according to Pfirrmann’s classification scores [37] based on the changes in the degree and the area of the signal intensity. All measurements were performed using the picture archiving and communication system (PACS) routinely used in the local hospital.

### 2.5 Tissue harvesting

At each of the three time points (1, 3 and 6 months), 6 rabbits were euthanized by an intravenous sodium pentobarbital overdose for histological and immunohistological analyses. The experimental IVDs (L2/3, L3/4, L4/5, and L5/6) were removed from each lumbar spine under sterile conditions. The specimens were then dissected sagittally and divided into two symmetric portions. Using one half of each disc (with the defect side) was used for histological analysis. From the other half of the samples (with the contralateral side), the nucleus pulposus (NP) was bluntly separated from the AF and then snap-frozen in liquid nitrogen. Samples were then stored at −80°C in preparation for s-GAG analysis.

### 2.6 Sulfated-GAG content measurement

NP samples were isolated from discs at each level at 6 months after surgery (*n* = 6). The PG content was quantified using the 1,9-dimethylmethylene blue DMMB assay [38,39]. Briefly, each lyophilized sample was digested with 125 μg/mL of papain (Sangon Inc., Shanghai; PRC) in sterile PBS, 5 mM of EDTA, and 5 mM of cysteine·HCl at pH 6.8 and 60°C overnight. After complete digestion, 20 μL of the papain digest was added to 200 μL of DMMB reagent (Sigma-Aldrich, St. Louis, Missouri, USA), and absorbance was detected at 520 nm. A Quant-iT™ PicoGreen® dsDNA Assay Kit (Invitrogen™) was used to determine DNA content in the discs, according to the manufacturer’s protocol (Thermo Fisher Scientific Inc., Waltham, MA; USA). The total sGAG in the disc for each group was normalized according to the tested amount of DNA, and the sGAG/DNA ratio was measured and reported.

### 2.7 Histology and immunohistochemistry

The specimens were fixed in 10% formalin, decalcified in ethylenediamine tetraacetic acid (EDTA), and processed for paraffin sectioning. Tissue blocks were paraffin embedded and sectioned at a thickness of 5 μm. Sections of the IVD samples were stained with hematoxylin/eosin (HE) to observe degenerative changes or with safranin-O/fast green to determine the PG content. Alternatively, the sections were subjected to immunohistological analysis for the nerve marker Protein gene product 9.5 (PGP9.5). CS-56 immunostaining was used to identify intact CS. All the stained sections were analyzed under an optical microscope (Leica Microscope, Wetzlar, Germany). Briefly, the epitopes for PGP9.5 immunohistology were first subjected to heat-induced retrieval. The sections were then blocked with hydrogen peroxide and 25% of normal bovine serum albumin (BSA)/tris buffered saline (TBS) and incubated overnight at 4°C with the primary mouse monoclonal antibody against human PGP9.5 (diluted 1:80, Abcam, Cambridge, GB). For CS-56 staining, endogenous peroxidase activity in the sections was blocked with 0.5% of hydrogen peroxide for 1 h, and the sections were then washed three times in TBS. Then, the samples were blocked with 25% of normal BSA for 1 h (both at RT), followed by an overnight incubation at 4°C with a primary anti-CS-56 mouse antibody (diluted 1:100, Sigma, St. Louis, MO). The sections were then washed again and incubated with a biotinylated goat anti-mouse IgG antibody (1:200; Vector Laboratories) overnight. The sections were then processed with the avidin–biotin amplification method with conjugated peroxidase (Vectastain Elite ABC Kit; Vector) and visualized with diaminobenzidine (DAB; Sigma). The sections were then counterstained with hematoxylin (blue) and mounted with Aquatex for observation under light microscopy. The control immunoglobulins consistently yielded negative results. The ingrowth of immunoreactive nerve fibers in the specimens was scored using a previously described grading scale [18,40].

### 2.8 Statistical analysis

Results are expressed as mean values ± standard error of the mean. Statistical analysis was performed using SPSS 22.0 software (SPSS Inc., Chicago, IL, USA). Significant differences in the radiograph measurements were analyzed by repeated-measurement analysis of variance (ANOVA) and Fisher’s least significant difference (LSD) test. The effect of time after surgery was analyzed with the Kruskal–Wallis test. Mann–Whitney *U* tests were used to analyze the MRI score, innervation, and biochemical data. The significance level was set at *p* < 0.05.


**Ethics approval and consent to participate:** All the procedures performed in this study involving animals were approved by the institutional review board and animal care committee of Tongde Hospital. The protocols were conducted in accordance with the Guidance for the Care and Use of Laboratory Animals, as formulated by the Ministry of Science and Technology of the People’s Republic of China and the “Principles of laboratory animal care” (NIH publication No. 86-23, revised 1985).

## 3 Results

### 3.1 PLGA/fibrin/ChABC morphology and ChABC release kinetics

**Figure 1:**
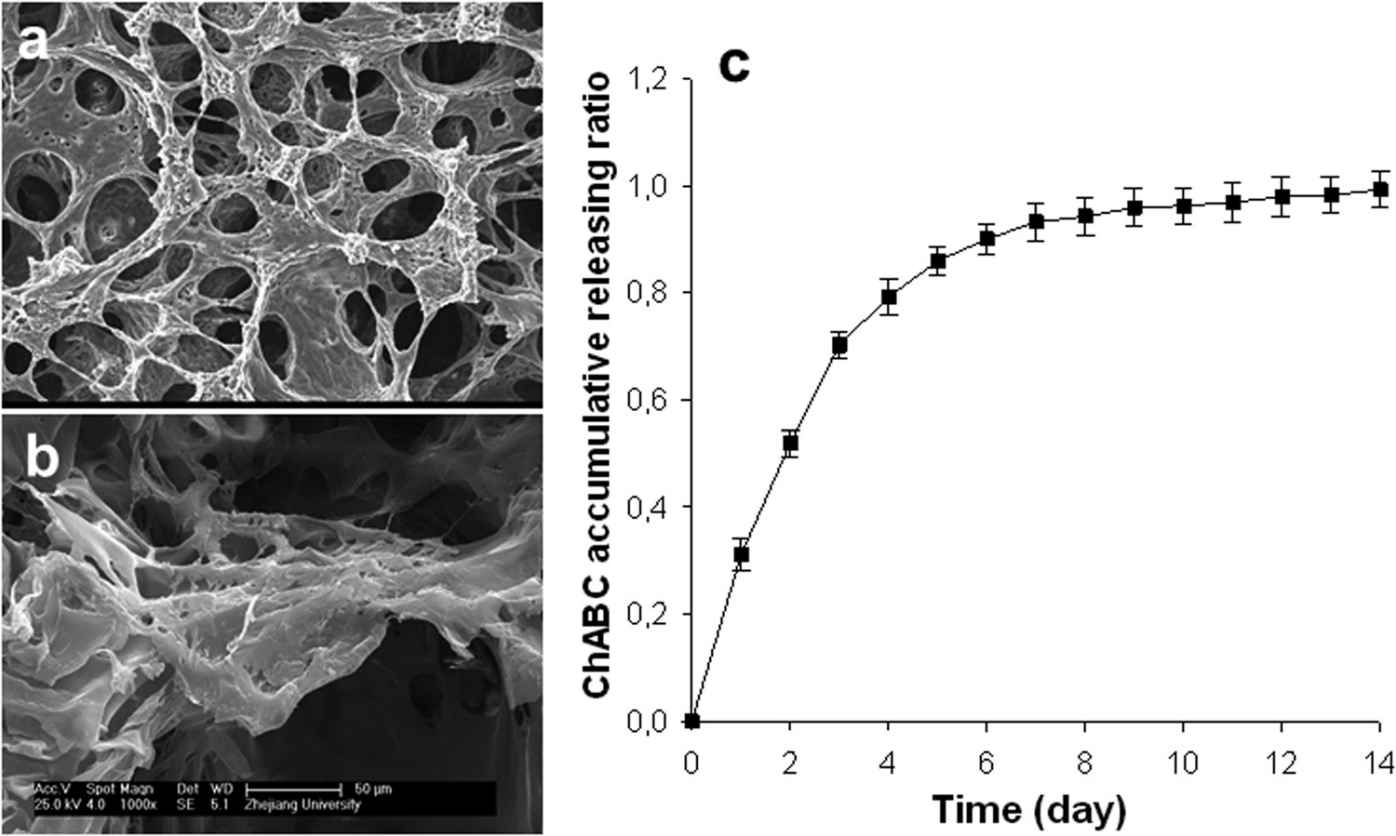
SEM micrographs of (a) the PLGA/fibrin gel sponge and (b) the composite sponge filled with fibrin gel and chABC at different magnifications; (c) *in vitro* cumulative release profile of chABC/PLGA/fibrin.

Fabricated PLGA sponges filled with fibrin gel and the chABC coating of the PLGA/fibrin construct were assessed by SEM and are shown in Figure 1a and b, respectively. The SEM micrographs revealed interconnected micropores with a mean pore size of 350 µm. The profiles of chABC release from the PLGA/fibrin scaffold over 14 days are shown in Figure 1c. The curve of chABC from fibrin-based carriers showed a significant initial burst release during the first 5 days, and the chABC release continued steadily thereafter. Overall, the cumulative release rate of active chABC reached approximately 80% after 8 days.

### 3.2 Radiographic and MRI assessment

**Figure 2:**
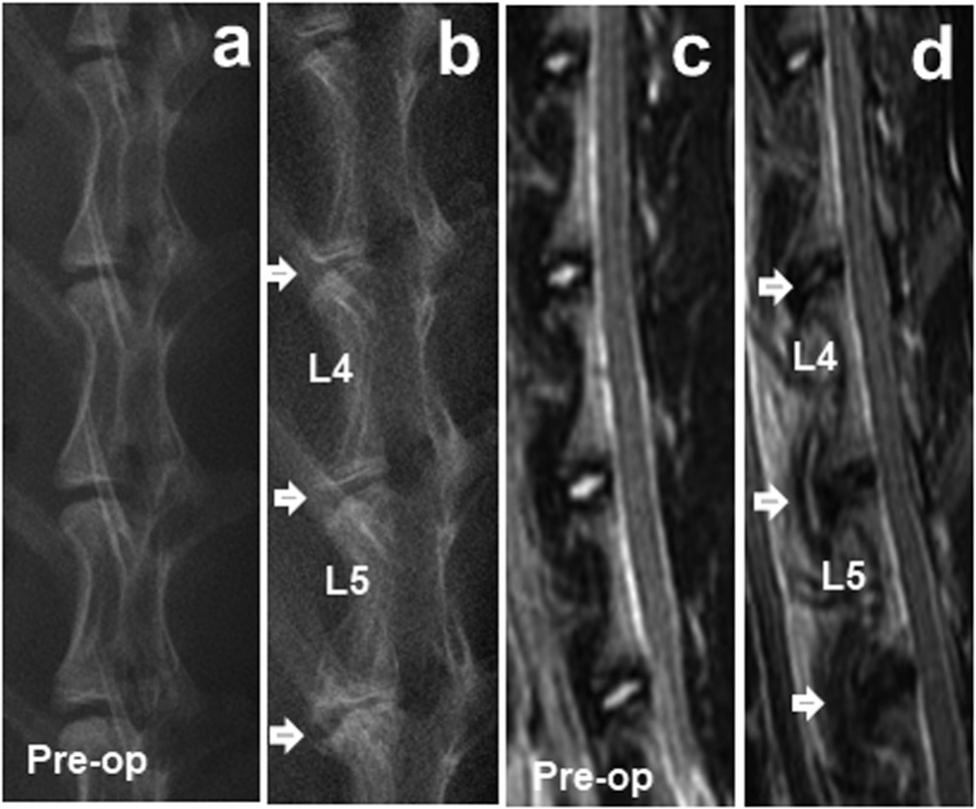
Representative lateral radiographs (a and b) and MRI (c and d) of the rabbit lumbar spine obtained 6 months after surgery. White arrows indicate that the injured disc received a different treatment.

**Figure 3:**
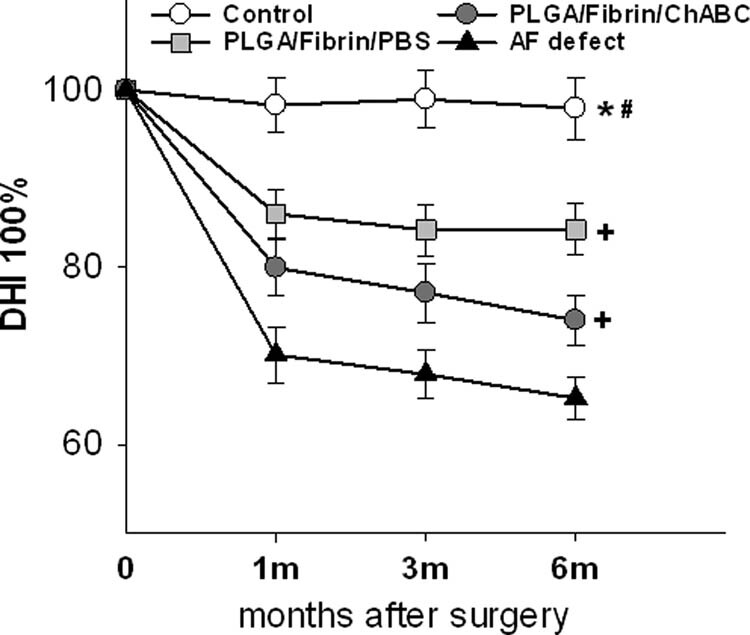
Changes in disc height index after surgery. There was a slow, progressive decrease in the disc height of operated discs over the follow-up period. The DHI values in both scaffold groups and the AF defect group were significantly reduced compared with that of the control group after surgery (*^,#^ *p* < 0.05, vs the control group). DHI% in the defect group was significantly decreased at 1, 3, and 6 months after surgery (^+^ *p* < 0.01, vs the two scaffold groups).

Compared with the normal control group, the disc height of the injured groups showed a slow but progressive decrease that was sustained for up to 6 months. At 6 months, a narrowing disc space and bridging osteophytes were observed in the two scaffold groups (Figure 2a and b). The DHI% of the two scaffold groups and the AF defect group were significantly reduced compared with those of normal control groups at all postoperative periods (*p* < 0.05), whereas no significant difference was observed between the PLGA/fibrin/PBS and PLGA/fibrin/chABC groups (*p* > 0.05). Notably, DHI% in the defect group demonstrated a significant decrease at 1, 3, and 6 months after surgery (*p* < 0.01) (Figure 3).

**Figure 4:**
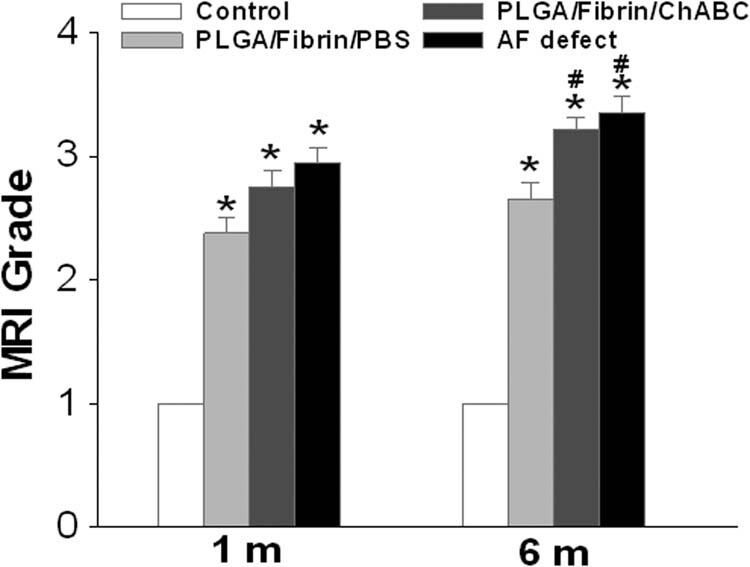
Changes in MRI grade after surgery. The MRI grades of the injured groups were progressively increased compared with those of the normal control groups at the postoperative time point (*p < 0.01, vs the control group). Both the MRI grades of AF defect and PLGA/fibrin/chABC discs were significantly increased compared with those of the PLGA/fibrin/PBS or control discs after surgery (^#^ *p* < 0.05, vs the control group or PLGA/fibrin/PBS group).

Serial MRI scans of rabbit lumbar spines revealed that the appearance of normal control discs remained relatively constant. Progressive decreases in the signal intensity in the NP area were apparent for each of the injured discs at all time points, as shown in Figure 2c and d. The MRI grades of the injured groups were progressively increased compared with those of the normal control groups at the postoperative time point (*p* < 0.01). Additionally, the MRI grades of the AF defect and PLGA/fibrin/chABC discs were significantly increased compared with those of the PLGA/fibrin/PBS or control discs after surgery (*p* < 0.05). However, no significant differences in the MRI grades were noted between the PLGA/fibrin/chABC and AF defect groups at 1 and 6 months after surgery (*p* > 0.05) (Figure 4).

### 3.3 Nuclear sulfated-GAG content

**Figure 5:**
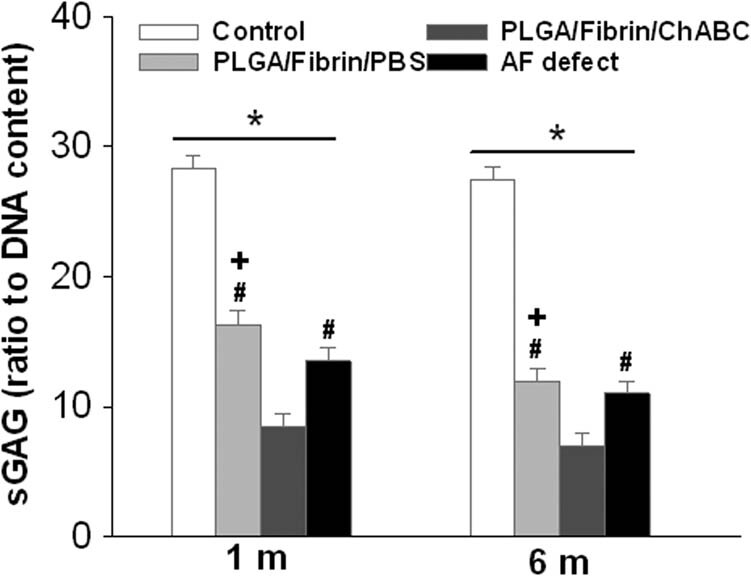
Changes in proteoglycan content of the NP at 6 months after surgery. The s-GAG content of the three injured groups decreased significantly compared to that of the control group at different postoperative time points (*p < 0.01, vs the control group). The PLGA/fibrin/chABC group exhibited a significant decrease in the sGAG/DNA ratio compared with the PLGA/fibrin/PBS or AF defect groups after surgery (^#^ *p* < 0.05 vs the PLGA/fibrin/PBS group or AF defect group). The decrease in the sGAG/DNA ratio observed in the AF defect group was significantly more pronounced than that in the PLGA/fibrin/PBS group at 1 and 6 months after surgery (^+^ *p* < 0.05).

The s-GAG content of the three injured groups decreased significantly compared to that of the control group at different postoperative time points (*p* < 0.01). The s-GAG content of the PLGA/fibrin/chABC group exhibited a significant decrease compared with that of the PLGA/fibrin/PBS and AF defect groups after surgery (*p* < 0.05). The decrease in the sGAG/DNA ratio observed in the AF defect group was significantly more pronounced than that in the PLGA/fibrin/PBS group at 1 and 6 months after surgery (*p* < 0.05) (Figure 5).

### 3.4 Histologic assessment

**Figure 6:**
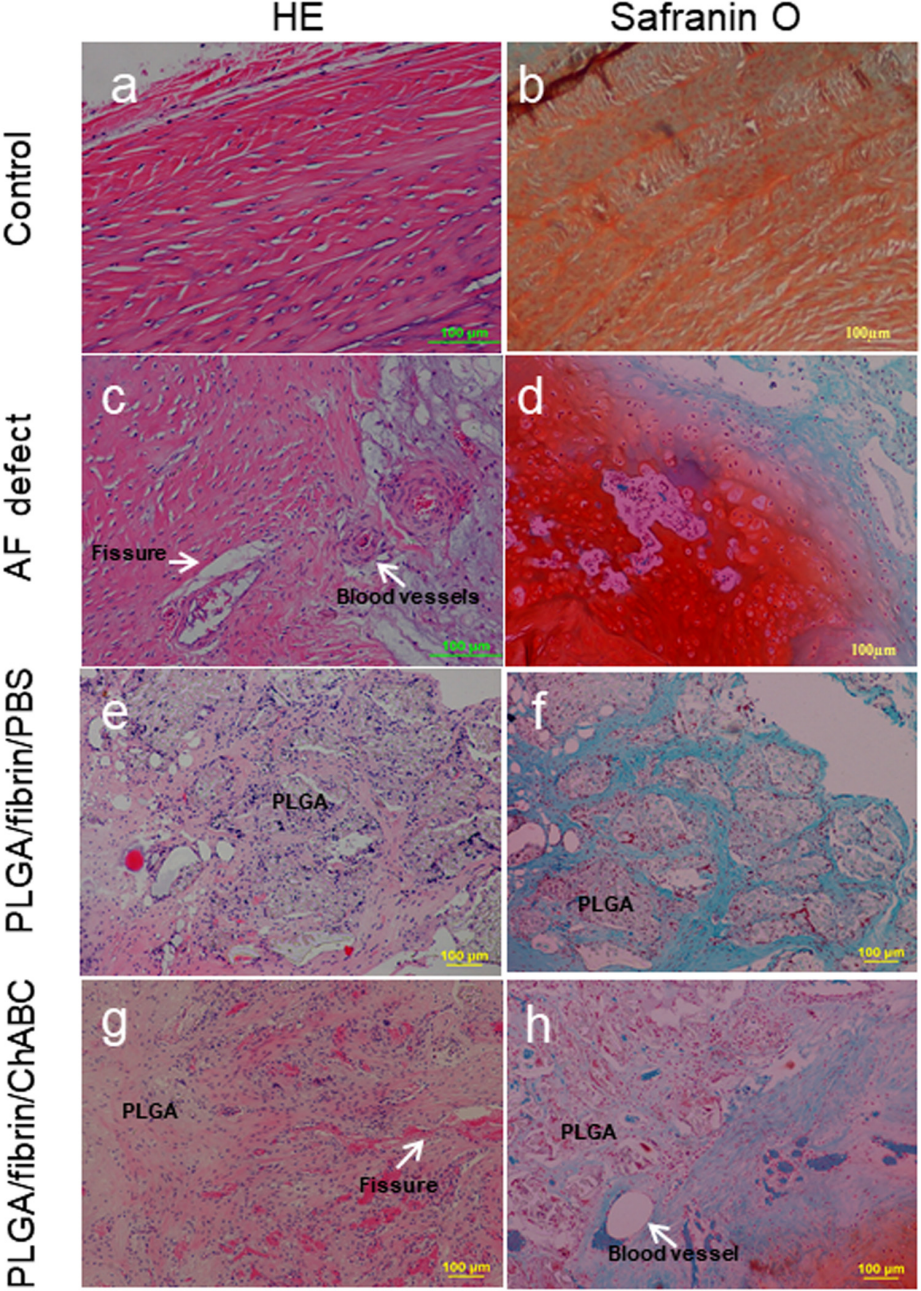
HE (a, c, e, and g) and safranin-O/fast green (b, d, f, and h) staining of the lesion site 6 months after surgery. The uninjured AF displayed a multilamellar structure rich in proteoglycans as demonstrated by strong safranin-O staining (a and b), loss of AF structural integrity, and replacement by extensive fibrocartilaginous-like tissue in the injured sites. Safranin-O staining indicated the presence of proteoglycan-rich content in the fibrocartilaginous tissue (c and d). Small irregular remnants of the PLGA scaffold were well integrated within the inner AF. Safranin-O staining revealed an open concave cavity with increased severe loss of the proteoglycan content (e and f). Advanced degeneration was observed in the PLGA/fibrin/chABC group, including NP fibrosis, disorganization of the AF, and reparative tissue extending further into the deeper inner AF along the fissures (g). The proteoglycan content was markedly reduced in the injured region (h). White arrows indicate areas of some blood vessels and small fissures.

At 6 months, HE staining demonstrated that the uninjured, healthy discs (control group) exhibited a characteristically well-organized intact AF with concentric lamellae. Upon safranin-O staining, uninjured discs exhibited a minimal disruption of the PG matrix within the annulus (Figure 6a and b). In the AF defect group, HE staining revealed the loss of the lamellated appearance of AF and the replacement of the annular defect by extensive fibrocartilaginous-like tissue that formed outside the injured sites. Some blood vessels and small fissures with a limited depth were typically distributed in the outer scar tissue and AF but did not extend to the inner AF (Figure 6c). Safranin-O staining indicated the presence of a PG-rich content in the fibrocartilaginous tissue (Figure 6d). In the PLGA/fibrin/PBS discs, HE staining revealed small residues of the PLGA scaffold with a naturally irregular form that were enfolded by newly formed tissue and well-integrated within the inner AF (Figure 6e). In addition, safranin-O staining revealed an open concave cavity with an increased loss of the PG content (Figure 6f). In contrast, advanced degeneration was observed in the PLGA/fibrin/chABC group, including NP fibrosis, disorganization of the AF, and new tissue clusters extending further into the deeper inner AF along the fissures. In addition, blood vessels and small fissures were commonly observed in the PLGA/fibrin/PBS group (Figure 6g). The PG content was markedly reduced in the injured region (Figure 6h).

### 3.5 CS-56 immunolabeling for CS-GAG digestion

**Figure 7:**
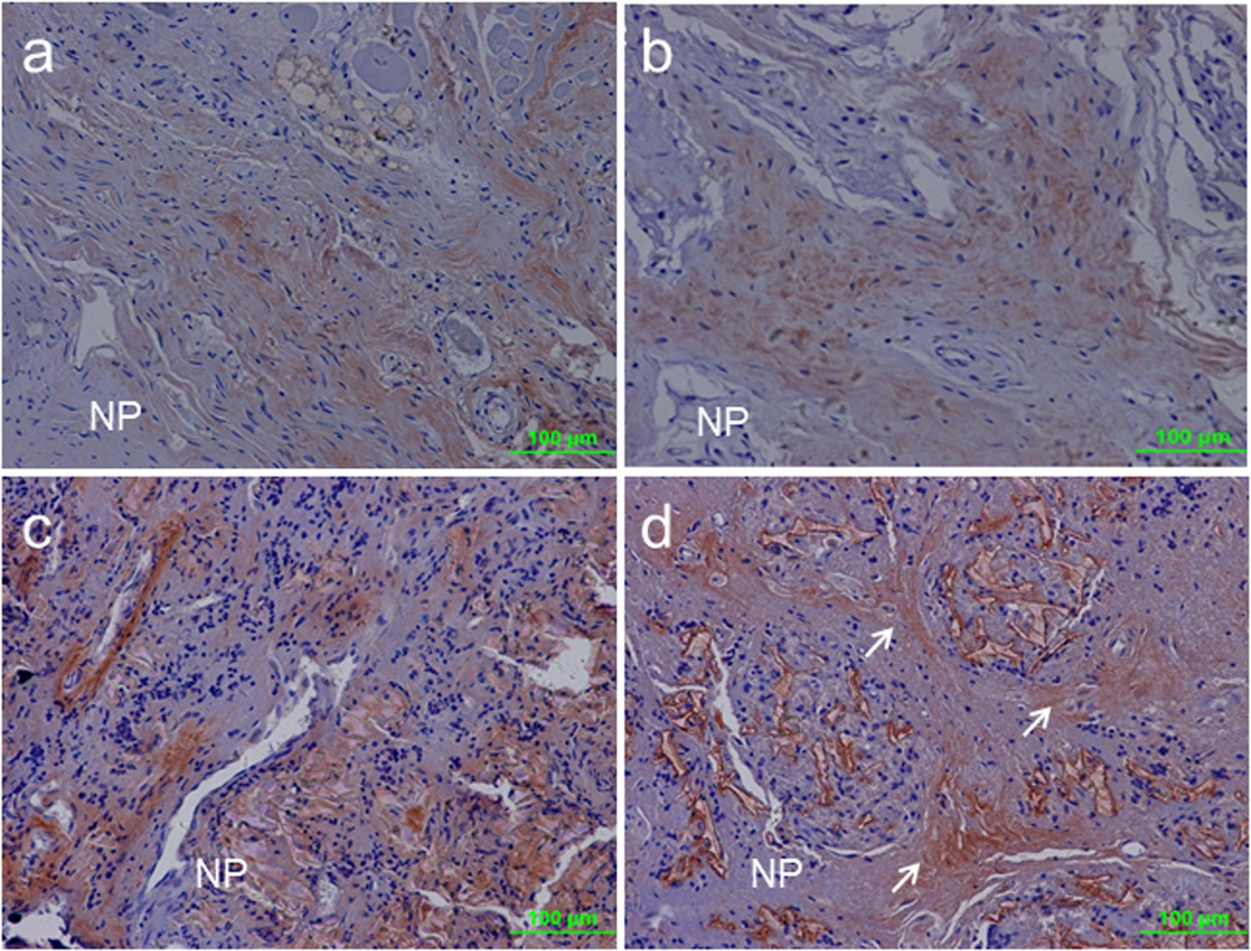
CS-56 immunohistochemical staining at the lesion site. (a) The outer lamellar AF was weakly stained with the CS-56 antibody in the control group. (b) In the injured discs, scar tissue formed on the surface of the injury site. A fuzzy CS-56-positive deposit was distributed in the scar tissue. (c) In the PLGA/Fibrin/PBS discs, newly formed tissue penetrated deeper toward the NP. CS-56-positive deposits were relatively negligible in the reparative tissue. (d) In the PLGA/Fibrin/chABC discs, CS-56-positive staining showed intense reactivity at the site of injury following treatment with chABC. White arrows indicate some regions of “intense” CS-56 reactivity. NP (nucleus pulposus).

CS-56 immunolabeling in the lesion site indicated that CS was degraded following treatment with chABC. In the control group, the outer lamellar AF was weakly stained with the CS-56 antibody (Figure 7a). In the injured discs, scar tissue was formed on the surface of the injury site. A fuzzy CS-56-positive deposit was distributed in the scar tissue (Figure 7b). In the PLGA/fibrin/PBS group, small residues of the PLGA scaffold were surrounded by newly repaired tissue penetrating deeper toward the NP. CS-56-positive deposits were relatively negligible in the clusters of the newly regenerated tissue (Figure 7c). In contrast, in the chABC/PLGA/fibrin-treated groups, CS-56 immunolabeling was high in the region of the newly generated tissue at the lesion site, but intense reactivity was noted overall (Figure 7d).

### 3.6 Innervation of the IVD

**Figure 8:**
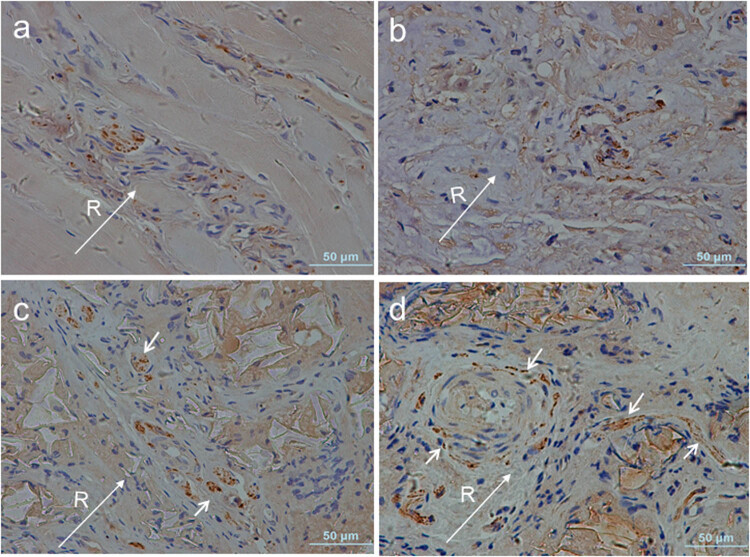
PGP9.5 immunohistochemical staining at 6 months after surgery. In the intact AF, PGP9.5-positive nerves were distributed sparsely in the lamellae and adjacent connective tissue of the outer AF (a). Small nerves in the vicinity of the outer AF were distributed along the fissures but rarely invaded the deeper inner AF in the AF defect group (b). PGP9.5-positive nerve fibers were present within the newly generated tissue of inner AF (c). In the PLGA/fibrin/chABC discs, more sprouting nerve fibers were predominantly identified in the vicinity of vascularized repair tissue and extended further into the inner AF (d). White arrows indicate areas of PGP9.5-positive nerve fibers. Long white arrows indicate the radial direction (from nucleus to outer annulus). R, radial.

PGP9.5-positive nerves were sparsely noted in the lamellae and adjacent connective tissue of the outer AF (Figure 8a). In the AF defect group, small nerves in the vicinity of the outer AF were distributed along the fissures, but these nerves rarely invaded the deeper inner AF (Figure 8b). In the PLGA/fibrin/PBS group, nerve fibers reactive for PGP9.5 were identified within the newly generated tissue of the inner AF (Figure 8c). In contrast, more sprouting PGP9.5-immunoreative fibers in the PLGA/fibrin/chABC group were localized predominantly in the vicinity of the vascularized repair tissue and extended further into the inner AF (Figure 8d).

**Figure 9:**
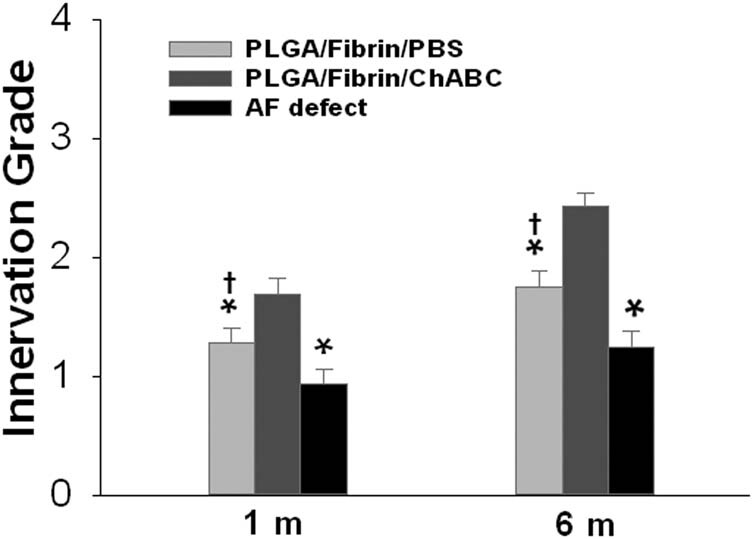
Changes in innervation scores at 6 months after surgery. The innervation score of the PLGA/fibrin/chABC group was significantly increased compared with that of the AF defect or PLGA/fibrin/PBS groups at 1 and 6 months after surgery (1 and 6 months, *p < 0.01). The innervation score of the PLGA/fibrin/PBS discs was significantly increased compared with that of the AF injured discs at 1 and 6 months (^†^ *p* < 0.05 vs the AF defect group).

The semiquantitative innervation scores were slightly increased in the three injured groups over the follow-up period. Notably, the innervation grade of the PLGA/fibrin/chABC group was significantly increased compared with that of the AF defect or PLGA/fibrin/PBS groups at 1 and 6 months after surgery (*p* < 0.05). The innervation score in the PLGA/fibrin/PBS group was significantly increased compared with that of the AF defect group at 1 and 6 months (*p* < 0.05), but no significant differences were noted among the different time points (*p* > 0.05; Figure 9).

## 4 Discussion

In our previous study [17,18,41], an AF injury model was established by creating a defect, which resulted in reproducible and degenerative MRI, radiograph, and histologic changes. Interestingly, degenerative changes occurred that were mainly characterized by regenerated tissue formation and the ingrowth of nerves and blood vessels into the disrupted disc tissue. More recent studies have demonstrated that aggrecan derived from both the AF and NP has inhibitory effects on nerve ingrowth into the IVD [13,18,42]. Thus, this study was conducted to evaluate the effects of degrading CS (CS)-PG on neurite ingrowth. Specifically, PLGA/fibrin gel with/without chABC was anchored in the annular defects during the degenerative process of the IVD. The results showed that fibrin-based PLGA loaded with chABC integrated well with the native annulus tissue and provided a local source for the sustained release of the active chABC. Importantly, more nerve fibers were orientated conductively to grow into the deeper PG-depleted regions of the injured annulus following chABC enzymatic deglycosylation. To the best of our knowledge, few reports have employed *in vivo* testing to assess the effects of PG-depleted sites in the annular wall on nerve ingrowth in a rabbit disc degeneration model.

It is well-known that chABC more specifically degrades the CS side chains of PGs [24,43]. Indeed, the use of a proteolytic enzyme for the induction of mild disc degeneration has been successfully achieved in both rabbits [44,45] and large sheep [19,33]. Furthermore, the reduction in PGs using a fibrin-based delivery system to achieve enzymatic digestion depends on its dosage and activity [25,46,47,48]. Based on the promising preliminary results, a dose of 0.1 U/mL of chABC incorporated into PLGA/fibrin gel was tested in the present study. Consistent with previous studies [23,34], the rate and duration of *in vitro* chABC release from the fibrin-based polymer were characterized by an initial burst (70–80%) that was sustained for up to 8 days. Thereafter, a small amount of chABC was still released from the PLGA/fibrin gel, achieving a delayed-release effect, as indicated in Figure 1c. In addition, CS-56 immunolabeling of the lesion site revealed that CS was locally degraded following treatment with chABC. Taken together, the results indicated that the administration of fibrin-based polymeric carriers at the injured site achieved sustained, localized release of chABC, and *in vivo* chABC activity was maintained after surgery, as demonstrated by the continuous significant decrease in the s-GAG content of the PLGA/fibrin/chABC discs compared with the two control discs. Conceivably, the present study offers a faithful analysis of the actual process of GAG reduction and its activity because it is very unlikely that the *in vitro* release kinetics would accurately reflect the more relevant *in vivo* release kinetics at the lesion site.

We observed PGP9.5-immunoreactive fibers in the defect group exclusively in the superficial area of scar tissue, and similar findings have been described in previous studies [18]. However, increased nerve ingrowth into PG-depleted discs sealed with PLGA/fibrin/chABC yielded a significantly increased innervation score compared to PLGA/fibrin/PBS or empty injured discs. In addition, this finding indicated that advanced degeneration with small fissures and vascularization occurs significantly more often and penetrates deeper into the PLGA/fibrin/chABC discs in the early postoperative period (Figure 6g). Eventually, the degenerative area appeared increasingly innervated in the chABC-treated discs following the reduction in PG. Furthermore, recent studies have shown that nerves and blood vessels grow into those regions of human lumbar discs that are damaged and depleted of PGs, supporting the present results from rabbit discs [12,15,49]. Several factors potentially influence nerve ingrowth into the injured IVD: (i) more extensive PG reduction in the depleted annulus regions is conducive to neural and vascular ingrowth following the chABC enzymatic deglycosylation; (ii) loss of structural integrity results in the early leakage of NP with the depletion of PGs, thus increasing the attraction of ingrowing nerves; (iii) the disruption of the tight collagen network that entraps the PGs eventually produces more new tissue (e.g., periannular innervated and vascularized granulation tissue) ingrowth along the local AF deficiency; and (iv) ingrowth is potentially inhibited by scar tissue due to the reduced PG loss compared with the newly regenerated tissue in the AF injured track. A further limitation of using enzyme degradation in an *in vivo* model involves the possibility that local undegraded islands of CSPGs may exist and are resorbed by cells synthesizing and replenishing the lost GAGs. Thus, these areas do not completely mimic a natural ‘degenerative’ process. Further studies are necessary to evaluate neural ingrowth in the degenerated IVD upon application of a control enzyme (e.g., hyaluronidase or matrix metalloproteinases) as a treatment option in a large animal model with longer follow-up times and dose-related deglycosylation.

## 5 Conclusion

Our results indicate that the chABC-based PLGA/fibrin gel showed promising results, achieving biointegration with native annulus tissue and providing a local source for the sustained release of active chABC. Disc PG-mediated inhibition of nerve and blood vessel ingrowth was abrogated by deglycosylation in an annular-injured rabbit disc degeneration model. Moreover, PG reduction may play a direct role in nerve and blood vessel ingrowth into degenerated IVDs. Intact GAGs, such as CS, secreted by disc cells are potential candidates that could be useful to reduce neural and vascular ingrowth associated with discogenic pain in degenerated IVDs.

## Abbreviations

AF: annulus fibrosus
ANOVA: analysis of variance
BSA: bovine serum albumin
chABC: chondroitinase ABCDMMB
CS: chondroitin sulfate
DHI: disc height index
DMMB: 1,9-dimethylmethylene blue
EDTA: ethylenediamine tetraacetic acid
GAG: glycosaminoglycan
HE: hematoxylin/eosin
IVD: intervertebral disc
LSD: least significant difference
MRI: magnetic resonance imaging
NP: nucleus pulposus
PBS: phosphate-buffered saline
PLGA: poly(lactic-*co*-glycolic acid)
PG: proteoglycan
PGP9.5: protein gene product 9.5
PACS: picture archiving and communication system
TBS: tris-buffered saline
